# Unleashing the power of anti-tumor CD4+ T cells: novel insights into the curative mechanisms of chemoimmunotherapy for cancer

**DOI:** 10.1186/2051-1426-3-S2-P18

**Published:** 2015-11-04

**Authors:** Tsadik G Habtetsion, Gang Zhou

**Affiliations:** 1 Georgia Regents University, Augusta, GA, USA

## Background

CD4^+^ T cells are critical mediators of anti-tumor immunity and orchestrate a broad range of immune responses against cancer. Previous studies from our lab and others have demonstrated that, adoptive transfer of tumor specific CD4^+^ T cells to lymphopenic hosts led to eradication of established tumors in mice models. Accumulating evidence from preclinical and clinical studies also suggest that CD4^+^ T cells in combination with chemotherapy can control tumor progression and recurrence. However, the molecular and cellular mechanisms by which tumor reactive CD4^+^ T cells eliminate a wide variety of tumors are not completely understood.

## Methods

In this project, we set out to study the mechanisms underlying the therapeutic effect of chemo-immunotherapy in the form of cyclophosphamide (CTX) and tumor specific CD4^+^ T cells. Recent studies have revealed that combined effect of Th-1 cytokines, IFN-γ and TNF, drive both murine and human cancer cells in to senescence. In the present study we wanted to examine the specific roles of IFN-γ and TNF-α in the setting of chemoimmunotherapy and the contribution of other immune cells in the tumor microenvironment to tumor rejection beside the donor CD4^+^ T cells.

## Results

In a mouse model of colorectal cancer, we found that host-derived interferon gamma (IFN-γ) and expression of IFN-γR are critical components of CD4^+^T cell-mediated tumor rejection, whereas depletion of NK cells and macrophages separately did not compromise the therapeutic effect of the CTX and CD4^+^T cells regimen. In addition, IFN-γ appeared to drive tumor senescence and apoptosis *in vivo*, leading to a curative outcome. Furthermore, we analyzed the global metabolic profiling of tumor tissues at different time points before and after chemoimmunotherapy.

## Conclusions

Our data suggests that CD4^+^T cells reprogram the metabolic profiling in tumor, tipping the balance towards progressive tumor regression. These findings may provide new insights into mechanisms of tumor rejection by CD4^+^ T cells, and may help develop more effective anti-tumor strategies based on a rational combination of chemotherapy and anti-tumor CD4^+^ T cells.

